# Nanofiber Based on Electrically Conductive Materials for Biosensor Applications

**DOI:** 10.1007/s44174-022-00050-z

**Published:** 2022-11-16

**Authors:** Seda Gungordu Er, Alesha Kelly, Sumudith Bhanuka Warnarathna Jayasuriya, Mohan Edirisinghe

**Affiliations:** 1 Department of Mechanical Engineering, University College London, Torrington Place, London, WC1E 7JE UK; 2 University of Chicago, Chicago, IL 60637 USA; 3 Department of Medical Physics and Biomedical Engineering, University College London, Malet Place, London, WC1E 6BT UK

**Keywords:** Nanofiber-based biosensor, Electrochemical, Electrospinning, Diagnose, Wearable, Implantable

## Abstract

Biosensors are analytical tools that enable the transmission of different signals produced from the target analyte to a transducer for the production of real-time clinical diagnostic devices by obtaining meaningful results. Recent research demonstrates that the production of structured nanofiber through various methods has come to light as a potential platform for enhancing the functionality of biosensing devices. The general trend is towards the use of nanofibers for electrochemical biosensors. However, optical and mechanical biosensors are being developed by functionalization of nanofibers. Such nanofibers exhibit a high surface area to volume ratio, surface porosity, electroconductivity and variable morphology. In addition, nanosized structures have shown to be effective as membranes for immobilizing bioanalytes, offering physiologically active molecules a favorable microenvironment that improves the efficiency of biosensing. Cost effective, wearable biosensors are crucial for point of care diagnostics. This review aims to examine the electrically conductive materials, potential forming methods, and wide-ranging applications of nanofiber-based biosensing platforms, with an emphasis on transducers incorporating mechanical, electrochemical and optical and bioreceptors involving cancer biomarker, urea, DNA, microorganisms, primarily in the last decade. The appealing properties of nanofibers mats and the attributes of the biorecognition components are also stated and explored. Finally, consideration is given to the difficulties now affecting the design of nanofiber-based biosensing platforms as well as their future potential.

## Introduction

With the recent technological advancements in diagnostic and therapeutic devices, tailored techniques that enable the identification of specific analytes have resulted in an increased need for analytical instruments [1, 2]. Accelerating the detection of biomarkers for certain diseases in individuals can make an important contribution to the improvement of wellbeing. Earlier diagnosis and monitoring tools increase the efficiency of treatment for serious diseases [3]. Biosensors are analytical tools developed to detect certain analytes or disease biomarkers used in clinical diagnoses. These devices provide large advantages for the user. The benefits include high sensitivity, high selectivity, cost efficiency, repeatability, and quick response. In general, the main elements of a simple biosensor comprise a bioreceptor for the recognition of analytes, a transducer for a physicochemical signal, and monitoring of results with data acquisition and processing. Electrospun nanofibers have been used as a key strategy for the production of biosensors in recent years [4, 5].

Nanofibers are one-dimensional nanomaterials with remarkable characteristics including high surface area, simple functionalization, controllable morphology and structure [6]. Nano-sized fibers are used in various fields such as wound healing, controlled drug delivery, air filtration and various biosensors within the healthcare industry [7]. The surface area to volume ratio of nanofibers is increased, as they typically have fiber diameters under 1 µm, frequently smaller than 500 nm [8]. In fact, this nanostructure, which occupies a relatively small volume, can contain a large number of fiber densities. The production and optimization of nanofibers have been the subject of several investigations.

Although electrospinning is the most common approach, the pressurized gyration technique [9], which was developed in recent years, has allowed manufacturing to be explored in both scientific research and industrial applications [10]. Both modes of production have an essential role in clinical research. The potential of using nanofibers has been investigated with the aim of building biosensors with larger loading capacity, greater sensitivity and selectivity, and rapid response time owing to their unique characteristics such as high surface area, porosity and immobilization. Nanofibers also enable the miniaturization of designed platforms [11]. The required biosensing qualities are also improved by straightforward functionalization, fiber surfaces and nanocomposites.

Physical surface modification methods such as electro air spraying, layer by layer, atomic deposition, and chemical methods such as oxidation, hydrolysis, grafting and cross-linking, and thermal methods such as calcination and heat press are utilized to improve the characteristics of nanofiber-based biosensors [12]. Aside from surface modifications, the materials used are intended to improve the sensing capability. Therefore, conductive polymers are generally preferred in the production of these nanofiber-based sensors. Conductive polymers are versatile materials and that are easily synthesized have desirable electrical and optical properties. These polymers have high electrical conductivity and examples are polyacetylene (PA) [13], polypyrrole (PPy) [14], polythiophene (PT) [15], polyaniline (PANi) [16], poly[3,4-(ethylenedioxy)thiophene] (PEDOT) [17]. These can be classified in a variety of ways based on their electrical charge, conductive nanomaterials, and ions [4, 18].

Biocompatible nanofibers are frequently produced using environmentally friendly, solvents with low toxicity such as acetone, ethanol, methanol, 2-propanol, ethyl acetate, isopropyl acetate, methyl ethyl ketone, and 1-butanol [19, 20]. Electrically conductive polymer nanocomposites are mainly composed of conductive nanofillers such as metal nanoparticles, metal oxides and carbon-based nanomaterials along with conductive polymer matrices. These nanocomposites have emerged as a prominent subject in the area of nanostructured fiber-based biosensors. These nanostructures are particularly preferred for improving electrochemical and electromechanical capabilities of biosensors, improving immobilization sites on the fiber surface, and increasing biorecognition [21]. However, in addition to these advantages, there are also certain limitations such as the high energy that may appear on the surface and the agglomeration of some nanoparticles in the solution [22, 23].

**Fig. 1:**
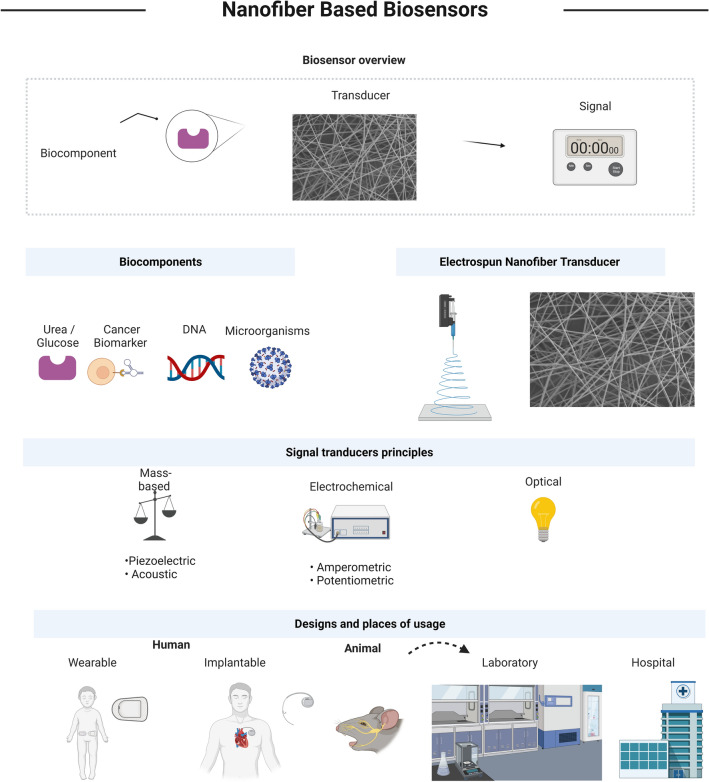
Illustration of nanofiber-based biosensor designs and main parts (All images within this figure are prepared in Biorender.com and have been used with permission from Biorender)

Recent research methodologies and designs of nanofiber-based biosensors are reviewed in this paper (Fig. 1). Biosensor applications are discussed in relation to the diagnosis of analytes, transducers, and other biomarkers or illnesses. Materials, nanomaterials, and techniques for functionalizing nanofiber biosensors are discussed in detail. Furthermore, successful applications are considered in accordance with design principles. The last section elucidates current limitations and future innovations.

## Nanofibers

### Conductive Polymers and Composites for Biosensor Applications

It is widely acknowledged that the 1977 paper reporting the doping of PA marked the beginning of contemporary research on electrical conductivity in conjugated polymers [13]. One of the most promising biocompatible materials is conductive polymers, which are natural conjugated polymers that conduct electricity and have a unique conjugated electron backbone system [24]. Polymer compounds known as conducting polymers have metallic and semiconductor capabilities, a unique mix of traits not shared by any other known material. Conjugated double bonds along the length of a conductive polymer's backbone are a crucial component. During conjugation, the backbone alternates between single and double carbon bonds between the atoms.

**Fig. 2:**
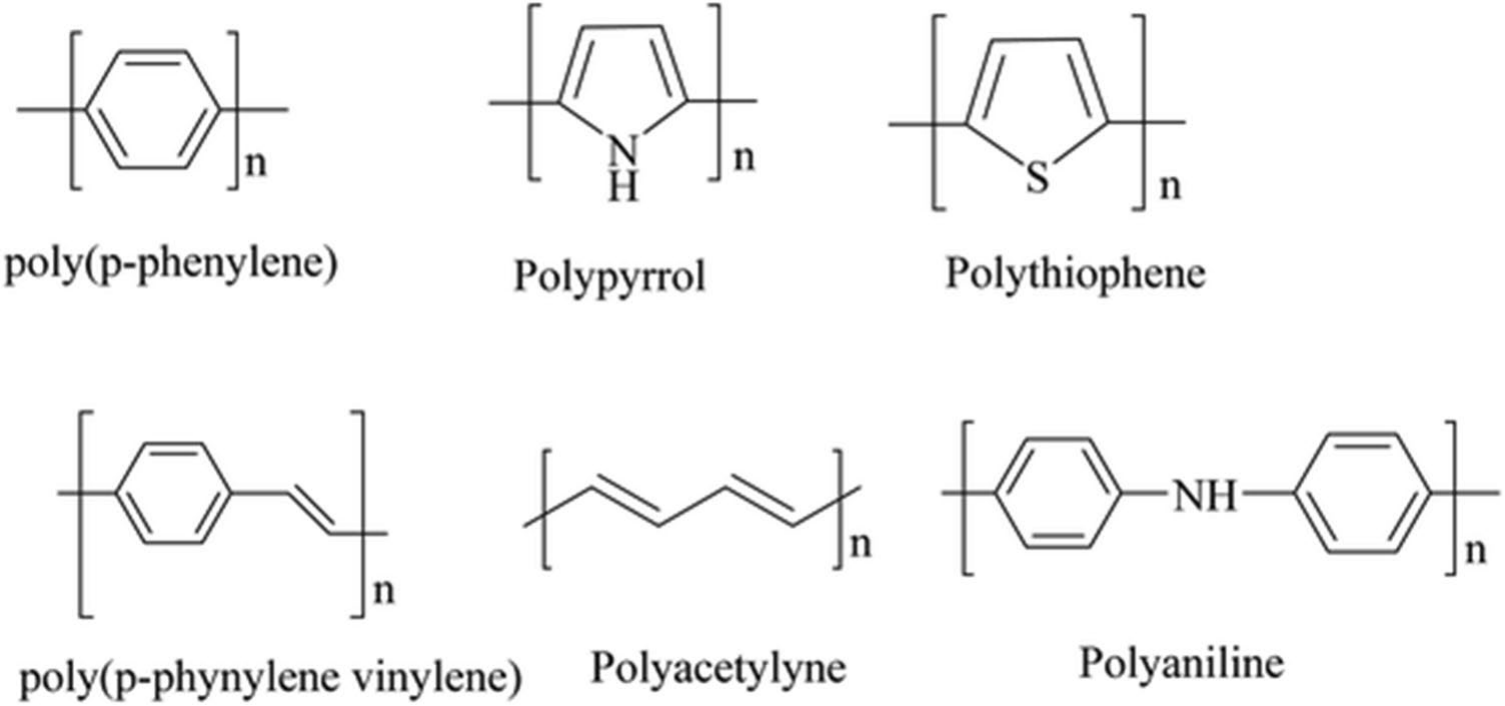
Electrically conductive polymers and their chemical structures Reproduced from Ref [26]. Copyright (2021), 11, 5659 with permission from the Royal Society of Chemistry

Conducting polymers, such as crystalline PA sheets combined with p-type dopants, were first discovered to have metallic conductivity by Shirakawa et al. in 1977 [13, 25]. These findings led to the establishment of a novel family of organic conductive polymers, also referred to as intrinsically conducting polymers (ICPs). The electrical conductivity of these polymers is facilitated by the presence of monomers that can acquire positive or negative charges through oxidation or reduction. ICPs are also known as conjugated π polymers and can include PA, PPy, PT, PANi, PEDOT (Fig. 2) [18, 26]. Conductive polymers can include ionic conductive polymers, redox polymers, and ICPs. The electro-conductive characteristics of redox and ionic polymers, however, are inferior to those of ICPs. The sensitivity is decreased in particular by the ease with which ambient temperature affects polymers that impact conductivity via ionic fluidity [4].

Conductive polymers have many desirable properties for use in electrospinning. This includes but is not limited to biocompatibility, ease of synthesis forming techniques, low cost, controllable conductivity over a large range and other electrical properties similar to that seen in metals and inorganic semiconductors [27]. These comparable properties allow for the possibility of conductive polymers to be used in place of metallic and inorganic conductors and semiconductors for biomedical applications [28]. This includes uses in biosensors, natural prostheses, wound healing, tissue engineering, and controlled release systems. Conductive polymers have been used in biosensors to capture biomolecules, made possible due to these polymers' superior properties [18]. Electrospinning has been used to produce nanofibers for use in biological sensors and tissue engineering, producing fibers that have a large degree of structural malleability and flexibility, unlike fibers produced from metals or inorganic conducting materials. Conductive polymer nanofibers can also be synthesized so that they are porous, resulting in them being permeable to molecules and solvents as desired [29].

ICPs have been widely utilized in biosensors as transducers, which operate as an intermediary layer between bioanalytes and the electrodes employed for signal monitoring. This is due to ICPs' capacity to transport electrons created by biochemical processes with high efficiency. They are also known to coexist in neutral aqueous solutions with biological molecules. ICPs have attracted a great field of interest as a promising material for entrapping biomolecules for the same reason. Numerous researches has investigated these distinguishing characteristics of ICPs to create a range of sensing devices for the identification of essential analytes important to a medical assessment [4]. The advantages of the biosensors include relative cost-effectiveness, conversable transduction of the signal, high sensitivity, and quick response times at ambient temperature [30].

The incorporation of functional nanomaterials into a polymer matrix can efficiently combine the advantages of each component, resulting in polymer composites with exceptional processability and a wide range of functions. These conductive polymer composites are produced by adding electrically conducting nanoparticles, such as carbon-based materials (graphene, graphene oxide), metallic nanoparticles, or metal oxides, into the insulating polymer matrix. This causes the polymer material to undergo an insulator to conductor transition. The conductive architecture in the polymer matrix has a considerable influence on the electrical and mechanical properties of conductive polymer composites (CPCs). Since the consistency of the nanofibers steadily improves as the filler content rises, the electrical conductivity often rises as well, resulting in an insulator-to-conductor transition of the CPCs. However, when the amount of filler materials is increased, the mechanical characteristics of the CPCs generally decrease. In addition to their electrical properties, CPCs' mechanical properties are crucial for application scenarios.

Due to the obvious connection between the conductive filler and polymer material, doping a high loading of conductive filler into polymer matrix considerably impacts the mechanical characteristics of the composites. Usually, compared to unreinforced polymers, the strength and stiffness are enhanced, but when the amount of nanoparticles increases, the elongation at break toughness usually suffers, which restricts the materials' application.

PANi, a conductive polymer, has gained significant interest due to its exceptional qualities, including its reversible and simple doping ability, customizable conductivity, and acceptable stability [31]. PANi can function as a self-contained mediator without the requirement of another mediator in the biosensor due to it having two redox couples [16]. Due to PANi's exceptional electrochemical properties and biocompatibility, PANi-based materials can be used in the detection of biological agents with rapid reaction speeds and with exceptional sensitivity. In comparison to its bulk, nanosized PANi has excellent sensitivity and a quicker response time since it has a reduced analyte penetration distance and offers a greater surface area [32].

Miao et al. reported the production of a glucose-sensitive biosensing device based on gold nanoparticle (AuNp)-polyvinylpyrrolidone (PVP) and conducting polymer PANi [33]. PVP was utilized, as a stabilizing and doping agent. Glassy carbon electrodes (GCEs) were used for the electrodeposition of these nanocomposites. The nanocomposite modified GCE's electrochemical and electrocatalytic characteristics and these aspects were investigated. The PANi-based glucose sensor features a broad linear range of 0.05 mM to 2.25 mM, a low detection limit of 1.0 × 10^–5^ M, 8 s of amperometric response time, and 9.62 μA mM^−1^ cm^−2^ sensitivity which is higher than the graphene-based biosensor electrocatalytic abilities (3,844 μA mM^−1^ cm^−2^) [34]. This sensor's outstanding stability and repeatability also made it possible to successfully detect glucose in samples of human serum. The increased surface area of the nanocomposite and the addition of more GOx are assumed to be the reason for the improved outcomes. In another study, Botewad et al. showed an evanescent wave absorption (EWA)-based straightforward, quick, and highly sensitive optical fiber urea sensor [35]. The suggested sensor displays a linear range of between 10 nM and 1 M, response time around the 50 s and a stable lifetime of 40 days. The created sensor is incredibly sensitive, trustworthy, and selective, with a lower detection limit of 10 nM. The sensing response varies for each urea concentration as a result of the PANi-ZnO modified cladding's altered optical and structural characteristics.

PPy is particularly attractive for industrial uses due to its high conductivity compared to many other conducting polymers, ease of production, and outstanding environmental stability [36]. As a conjugated polymer, it has the ability to alter volume and produce large stresses and strains. It is possible to pattern these materials using traditional microfabrication methods. The in situ synthesis of the PPy-silver (Ag)-PVP nanohybrid is shown in a study using AgNO_3_ as an oxidant and PVP as a stabilizer and surface-active agent [37]. Additionally, the synthesized PPy-Ag-sensitive PVP and specific dopamine sensing have been investigated. With incredible sensitivity (7.26 μA mM^−1^ cm^−2^), the detection limit is determined to be 0.0126 μM. Dopamine concentrations in human urine samples from various age groups have been used to validate the practice implementation of the current modified electrode [37].

It is still extremely difficult to develop a novel method that is both reliable and cost-effective for detecting uric acid without the use of uricase. According to Wang et al., a monolithic peroxidase mimic is encapsulated in polyoxometalate and coated with PPy, making it simple to realize, affordable, and uricase-free for selective colorimetric biosensing of uric acid [38]. These findings lead to the invention of a uricase-free colorimetric biosensing instrument for uric acid, which has a wide linear detection of 1–50 μM and a low detection limit of 0.47 μM. More notably, this proposed biosensor is well suited for straightforward and accurate uric acid identification in biological materials, indicating strong application potential in clinical diagnostics and associated domains [38].

PEDOT, well known as the most effective conducting polymer, plays an important role due to its superior film-forming abilities, high transparency, adjustable conductivity, thermal stability, water-processibility, and high flexibility [39]. Cetin et al. offered an inexpensive method for making PEDOT nanofiber biosensors utilizing straightforward methods [17]. The loading of varying quantities of GOx onto the nanofibers enabled the creation of biosensors. The 0.6 V biosensor displayed a large linear range, improved stability, superior precision, and low limit of detection (2.9 μM) and a quick response time of around 2-3 s. The PEDOT-NFs/GOx-2 biosensor's improved sensitivity was 272.58 μA mM^−1^ cm^−2^ when it was functioning at 0.6 V and PEDOT-NFs/GOx-3 had sensitivity around 74.22 μA mM^−1^ cm^−2^.Their research has demonstrated that these interference-free, simple-to-build sensors offer strong prospects for commercialization since they have high sensitivity and stability [17].

On the other hand, Saunier et al. have developed an amperometric biosensor to improve the performance of neuronal therapies on the market using a PEDOT: cellulose nanofibrils (CNF) hybrid nanostructure [40]. In this study, the large surface area and loading capacity of this material increase the reliability of the nanoplatforms containing the PEDOT polymer. In addition to the impressive electrochemical results for serotonin and dopamine neurotransmitters, it produced an effect with its low specific impedance in contrast to similar organic materials produced in the same period. The results were linear concentration of 0.1 to 9 μM and 0.06 to 9 μM, high sensitivities of 44.54 pA nM^−1^ m^−2^ and 71.08 pA nM^−1^ m^−2^ and low detection limits of 0.045 μM and 0.056 μM, respectively. Cell viability and cytotoxicity of this composite material coated microelectrodes were assessed to demonstrate that this novel material did not promote any toxic effect. These properties show a promising structure for other unique materials in neuronal therapies and therefore structural manipulation is a key strategy here.

### Forming of Nanofibers

Many methods are available but two main techniques which can be scaled up for mass production are briefly described below.

#### Electrospinning

**Fig. 3:**
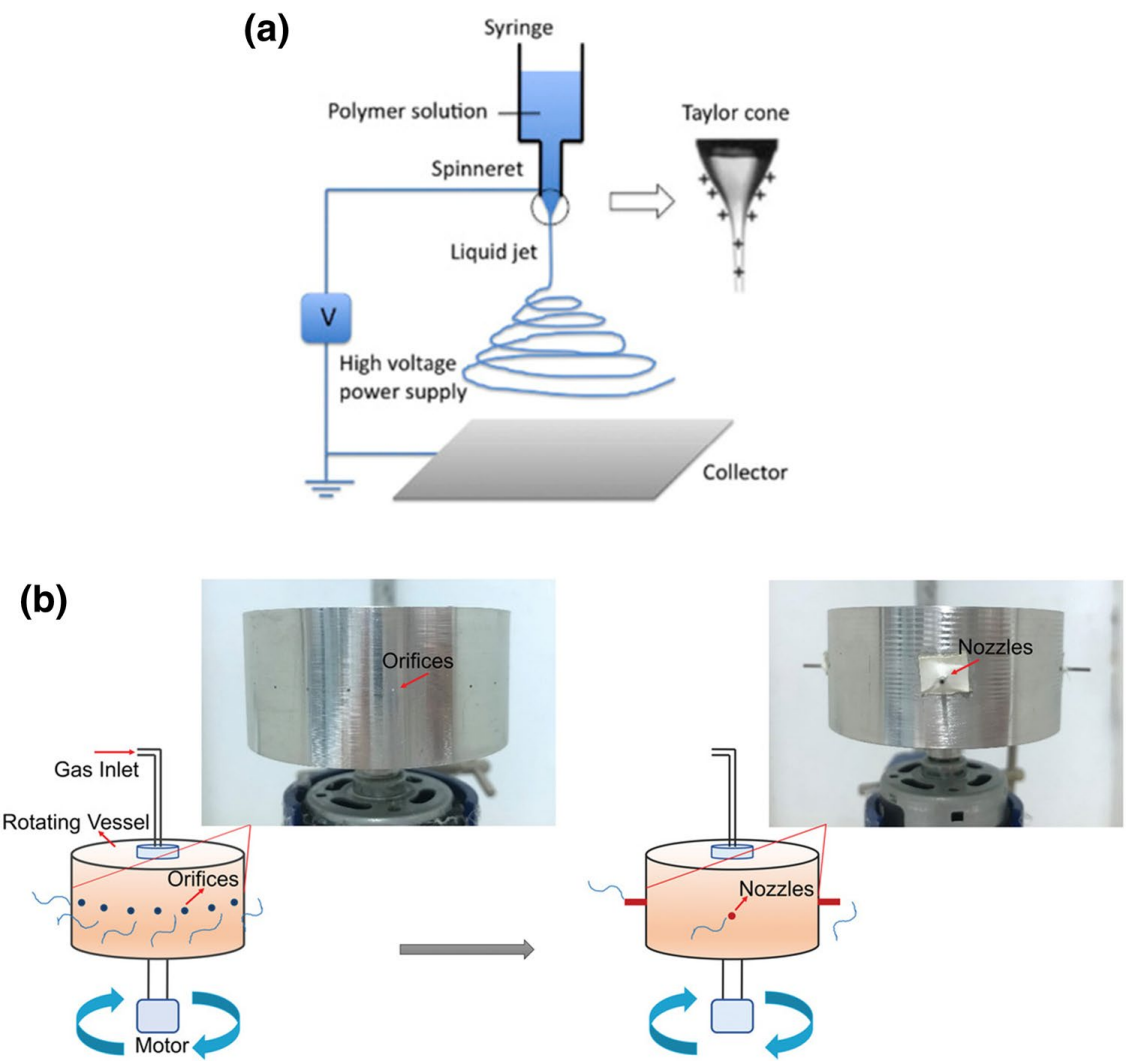
**a** Electrospinning illustrations Reprinted from, Ref. [53]), with permission from Elsevier Copyright (2015). **b** Pressurized gyration, nozzle pressurized gyration Reproduced with permission of Ref. [50] Macromolecular Materials Engineering Copyright (2022) 2200268

Electrospinning has a wide range of usage in the production of nanofibers for application in many fields [41]. This process uses electrostatic forces to form fibers from a solution as it solidifies. Provided the solution is continuously supplied, uninterrupted fiber production is achieved from the solution jet [42]. As shown in Fig. 3a, a drop of polymer solution at the tip of the nozzle is contorted into a conical-shaped nozzle (Taylor cone) from which a fine jet of polymer solution is ejected provided the supplied voltage surpasses the threshold required to overcome the surface tension of the polymer solution [43]. A high voltage power supply is used to accelerate the selected polymer solution towards a grounded collector which normally has an opposing polarity to the needle.

This method can produce fibers with diameters ranging from nanometers to micrometers at ambient temperature and pressure [44]. The diameter and morphology can be altered through the adjustment of the parameters of the experimental setup. This includes variations in the collection distance, the applied voltage, and the flow rate of the polymer solution. Furthermore, changing the parameters of the polymer solution used, such as the electrical conductivity of the solution, the volatility of the solvent and the concentration of polymer in the solution, can also affect the morphology of the fibers obtained [8]. Subsequently, fibers can be produced having a small diameter and increased surface area to volume ratio allowing for wide applications within the biomedical field, including uses in drug release, tissue engineering and wound dressing [45, 46].

Electrospinning is a method that has a simple process, especially for forming of nano-sized fibers. In this way, it allows obtaining the desired structure for many areas such as antimicrobial filtration and biosensing with its high surface area to volume ratio properties. In addition, ease of control and cost-effectiveness are among its important advantages. However, while scientific research can be possible in laboratories with electrospinning, mass productive by electrospinning to industrial scale is not straightforward. Also, the use of high voltage power supply may pose a danger in terms of the user but the small current encountered mediates this danger somewhat. In addition, the power supply used can negatively affect the formation of electroconductive nanofillers and fiber structure.

#### Pressurized Gyration

Pressurized Gyration uses a cylindrical aluminum vessel with perforations to produce fibers. An attached motor is used to spin the vessel which is connected to a gas outlet with the ability to vary the flow pressure [47]. The centrifugal force created as the vessel rotates as well as the selected pressure forces the polymer solution out through the perforations, creating a polymer jet that can be collected as fibers once the solution has dried [48]. The diameter of the fibers collected (ranging from 60 to 1000 nm) is dependent on parameters selected during fiber production. Variation in the concentration of polymer solution, selected working pressure, and the speed of rotation all affect the diameter and morphology of the fibers obtained.

The length of the collected fibers can also be varied through changes in the rotational speed [9]. As the fiber morphology can be altered to reduce the diameter and increase surface area, the produced fibers can be used for a wide range of applications in the biomedical science and engineering. This includes uses in tissue engineering, drug delivery, filtration, hydrogels and wound healing [10]. These applications are further extended by the development of core-sheath nanofibers used for the drug delivery [49] and nozzle pressurized gyration [50] (Fig. 3b) where uniform and aligned nanofibers are able to be layered within each fiber strand [51]. This allows for the development of novel structures and materials for utilization in many biomedical scenarios [52]. Although pressurized gyration has not been specifically used to date to make nanofiber applied biosensors, it offers promising potential as a serious alternative to electrospinning.

Pressurized gyration provides safer fiber production since no voltage is applied. At the same time, it provides a significant advantage for the high-amount production for industrial applications. However, it is difficult to control and its output units the micro-scale more.

Sometimes, the production methods of nanofibers may not provide the nanofibers with sufficient properties for certain applications and in these cases, these nanofibers need to be improved and modified. Post-surface modification methods [12] used are presented as promising alternative methods in biosensor applications. This post-functionalization of nanofibers can enable nanomaterials used in nanofiber hybrids to improve nanofibers for biosensing. These methods are included in research with many different techniques incorporated physical, chemical, and thermal principles. It has been observed that dip coating and spray coating methods are used especially in biosensor applications [54, 55].

Ulker et al. produced nanofibers from iron oxide—silk fibroin nanocomposite by electrospinning method [54]. Firstly, the encapsulation method was applied, and secondly, the dip coating method was used for the modification of the surfaces of the nanofibers. While nanomaterials at different concentrations were coated with silk fibroin with the first method, they were promising for tissue engineering applications, while the second method offered a suitable surface for the biosensor for dip coating application in different solutions. In another study, carbon nanotubes were used to coat electrospun nanofibers [55]. This spray coating was applied to improve the electroconductive properties of PU nanofibers. When the results were analyzed, it was observed that the coating on the nanofiber mat increased the electrical conductivity. In addition, it has been reported that increasing the spray frequency decreases the electrical resistance and improved surface can be useful for biosensing applications.

## Fibers as Physicochemical Transducer

### Electrochemical

Electrochemical transduction setups typically involve three electrodes, namely, the working, counter, and reference electrodes. The working electrode is where the biorecognition phase of biosensing occurs. The reference electrode provides a stable reference potential. The counter electrode is used to complete the circuit by allowing the current to flow. Various electrochemical measurement approaches including but not limited to potentiometry, voltammetry, amperometry, and impedometry can be carried out to determine analyte concentration.

Amperometry and voltammetry entail the application of a static or varying potential across the working electrode with respect to the stable reference electrode [56]. Consequently, a redox reaction takes place at the working electrode, where an oxidizing substance loses electrons and a reducing substance gains electrons. In the context of biosensing, the target analyte whose concentration/presence is being measured is in some way involved in the redox reaction. Amperometry and voltammetry both include measuring the current, which is dependent on the flow of charges due to the redox reaction occurring at the working electrode. As the extent to which the redox reaction transpires relies on interactions involving the target analyte, such measurements can be used to derive information about the analyte concentration. In potentiometric measurements, changes in the potential difference between the working electrode containing the recognition element and the stable reference electrode, when there is no current passing through the circuit are monitored [56]. The changes in potential are logarithmically related to analyte concentration according to the Nernst equation. The principle behind impedometry is that the extent of electron transfer between the sample being investigated and the electrode surface decreases when the analyte binds to the electrode surface, which leads to an increase in electrode impedance [56].

**Fig. 4:**
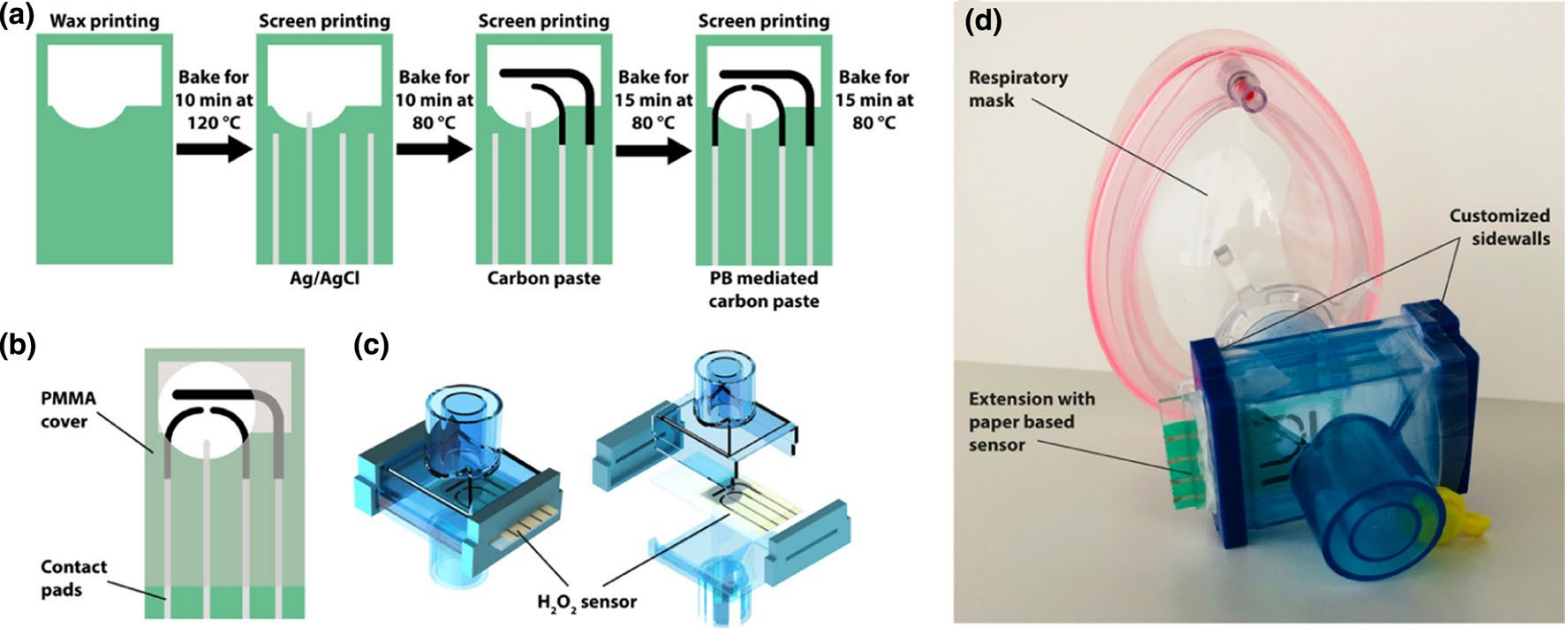
**A** Ag/AgCl, Prussian Blue (PB), carbon mediated electrode chip design stages. **B** CAD drawing of the sensor containing polymethylmethacrylate (PMMA). **C** Solid work design model paper based hydrogen peroxide (H_2_O_2_) sensor. **D** Paper-based electrochemical respiratory mask Reprinted from, Ref [62], with permission from ACS Sens. Copyright (2019)

By measuring electrode impedance and conducting suitable calibration, the increase in impedance can be related to the concentration of analyte in the sample solution. Based on the approaches mentioned above, electrochemical transducers can convert the biochemical effects caused by interactions between the analyte and the bioreceptor on the working electrode surface into a measurable electrical signal. As demonstrated in a review by Halicka and Cabaj [11], nanofiber-based biosensors have been developed to utilize electrochemical transduction to detect a range of analyte types. These include; biomolecules like glucose, dopamine, and progesterone; pharmaceutical drugs like penicillin and morphine; metal ions like mercury and arsenic ions [11]. As evident in several studies [57–61], Ag|AgCl is commonly used as a reference electrode (Fig. 4) [62]. In addition, the working electrode in nanofiber-based biosensors typically comprises some variation of nanofibers, which can have a multitude of surface-bound biorecognition elements.

### Optical

Optical transduction can be utilized when interactions between an analyte and a corresponding biorecognition element result in an observable optical phenomenon [11]. Optical transducers can convert optical phenomena generated during biorecognition into electrical signals. The strength of this observable effect, and in turn the strength of the transduced signal is associated with the analyte concentration. Optical transducers can employ various techniques such as measurements of absorbance, fluorescence, colorimetry, luminescence, or scattering of light, all of which are influenced by analyte concentration. Absorbance measurements can be used for optical transduction by measuring absorbance before and after the sensing surface has made contact with the target analyte. The increase in absorbance can be used to quantify the amount of analyte present in a sample. Fluorescence-based optical transducers operate on a similar principle where the fluorescence before and after the analyte-recognition element interactions occur.

Colorimetry is another popular avenue for optical transduction, where the wavelengths and intensity of visible light are measured prior to and following analyte adsorption onto the sensing surface. The absorbed wavelengths and the decrease in intensity of the visible light due to the analyte provide information about the presence of a specific analyte, as well as the concentration of the analyte. Other noteworthy approaches used for optical transduction include surface plasmon resonance (SPR) and its variations, ellipsometry, optical waveguide interferometry, and reflectometric interference spectroscopy. Surface plasmon resonance is a particularly well-known technique for optical transduction. The underlying physics on which this technique is based is that surface plasmons are produced when a conducting surface (usually a metal) present at the boundary between two optically different mediums is illuminated by polarized light. This in turn causes changes in the refractive index of the surface. When the incident polarized light is at a specific angle (the resonance angle), the generation of plasmons brings about a reduction in the intensity of the light reflected from the surface. The position of the reduced intensity beam which reaches the optical detector is dependent on the amount of analyte present at the surface. Once again, the review by Halicka and Cabaj [11] highlighted several examples where optical transduction-based nanofiber biosensors were developed to detect a myriad of analytes ranging from metal ions like mercury, iron, and copper ions [63–65], to biomolecules like thrombin, dopamine, and riboflavin [66–68].

### Mechanical

Mechanical transducers can be implemented in biosensing applications where interactions between analytes and biorecognition elements result in mechanical responses such as changes in force, displacement, and mass [69]. These mechanical changes can be quantified and used to produce electrical signals with values dependent on the extent to which biorecognition occurs. Perhaps the most distinguished mechanical transduction method involves the use of cantilever probes, which are usually on the scale of micrometers or nanometres. The cantilever probes can be operated in two modes, namely, the static and dynamic modes. In the static mode, attachment of the analyte onto either surface of the cantilever mode results in surface stresses acting on the cantilever, which causes deflection or strain of the cantilever. The extent of the displacement of the cantilever can be measured and related to the amount of analyte present on the surface. The methods commonly used to measure the deflection of the cantilever include the use of piezoresistive sensors [70], as well as laser beams reflected off the surface [71].

It is worth noting that in the static mode, long and flexible cantilever probes are desirable to maximize deflection [70]. In contrast to the static mode, actuation of the cantilevers is necessary in the dynamic mode to reach the resonant frequency [70]. Mechanical transduction in this mode entails the measurement of the change in the resonant frequency of the cantilever upon analytes binding onto its surface. The change in the resonant frequency is related to the amount of analyte on the surface. For the dynamic mode, shorter and stiffer cantilevers are preferable due to higher resonant frequencies, making the measurements less susceptible to low-frequency noise from the surroundings. Another prominent approach used for mechanical transduction is piezoelectric. In this method, acoustic waves are produced by an oscillating piezoelectric crystal [72]. When an analyte binds to the surface, the frequency at which the piezoelectric material oscillates, and in turn the frequency of the acoustic wave decreases [73]. The magnitude to which the frequency decreases is related to the amount of analyte.

## Target Bioanalyte

There are numerous distinct biorecognition components (Table 1) such as DNA, protein, cancer biomarker, cardiac biomarker, microorganisms, glucose, urea each with its special characteristics. Many different biosensing platforms have been developed for the detection of these bioanalytes.

**Table 1:** Summary of nanofiber-based biosensors, all have an electrochemical transducer and in the case of Refs. [74] and [85] they were also impedimetric

Nanofiber composites	Analyte	Sensing Parameter	Ref
Polyurethane/Poly(m-anthranilicacid) (PU/P3ANA)	DNA-*Salmonella*	Linear range of 1 μM and 10 μM, selectivity 8.17 kΩ μM^−1^	[74]
Cellulose monoacetate/Nafion (CMA/N)	DNA	–	[75]
Cellulose nitrate-PANi	*E. coli*	Linear range of 10^1^–10^4^ CFU mL^−1^ Sensitivity 67 CFU mL^−1^	[76]
PAN-Copper (Cu)-doped (Zinc Oxide)ZnO	HRP2 protein *Malaria* parasite	Linear detection ranges of 10 ag mL^−1^ to 10 μg mL^−1^, Sensitivity 28.5 kΩ (gm mL^−1^) cm^−2^	[78]
Carbon nanofibers	*Hepatitis B virus*	Linear range of 1 × 10^–12^ to 1 × 10^–6^ M, sensitivity 1.58 × 10^–12^ M	[79]
PEDOT-poly(4-styrenesulfonate)(PSS)-PVA	Carcinoembryonic antigen (a cancer biomarker)	Linear range of 0.2–25 ng mL^−1^, sensitivity 14.2 μA ng^−1^ mL cm^−2^	[81]
Multi-walled carbon nanotubes (MWCNTs)	Cardiac biomarker	Detection limits of 6, 20 and 50 fg mL^−1^	[82]
Cellulose acetate (CA)-rGO	Glucose	Linear range of 3.3–27.7 mM Sensitivity 9.9 × 10^–4^ kΩ^−1^ mM^−1^	[83]
Nanoporous gold (NPG)	Glucose	Linear range of 0.01–1 mM Sensitivity as high as 253.4 μA cm^–2^ mM^–1^	[84]
PAN/PPy/poly-pyrrole-3-carboyxylic acid (PPy3COOH)/glucose oxide (GOx)	Glucose -urea	Linear range of glucose concentration (20 nM–2 μM)	[85]

### Protein and Microorganisms

Gokce et al. developed a platform that enables *Salmonella* bacteria to be recognized with an impedimetric DNA biosensor. This biosensor contains an intermembrane transducer structure with electrospun nanofibers [74]. With Polyurethane/Poly(m-anthranilic acid) (PU/P3ANA) nanofibers, the DNA biosensor showed good selectivity and sensitivity parameters, while the linear response was between 0.1 and 10 μM, the selectivity was specified to be 8.17 kΩ μM^−1^. This research enabled a nanofiber-based impedimetric DNA biosensor to recognize *Salmonella* species and showed potential implications in terms of other microbes. In another study, it was stated that electrochemical biosensors for DNA bioanalyte showed biosensor properties [75]. Nanofibers used as interfaces in this study enabled cellulose monoacetate/Nafion (CMA/N) composites to be brought together and shaped by electrospinning. The fiber diameter distribution was observed to be between 500 and 1.5 μm. In addition, it has been reported that this nanostructure placed on the spherical graphite electrode supports biosensing properties. Nafion hybrid modified DNA molecules were immobilized on the obtained nanofiber-based biosensor and their electrochemical properties were investigated by differential pulse voltammetry (DPV). According to the results, it was stated that this platform can be used as an electrochemical DNA biosensor.

**Fig. 5:**
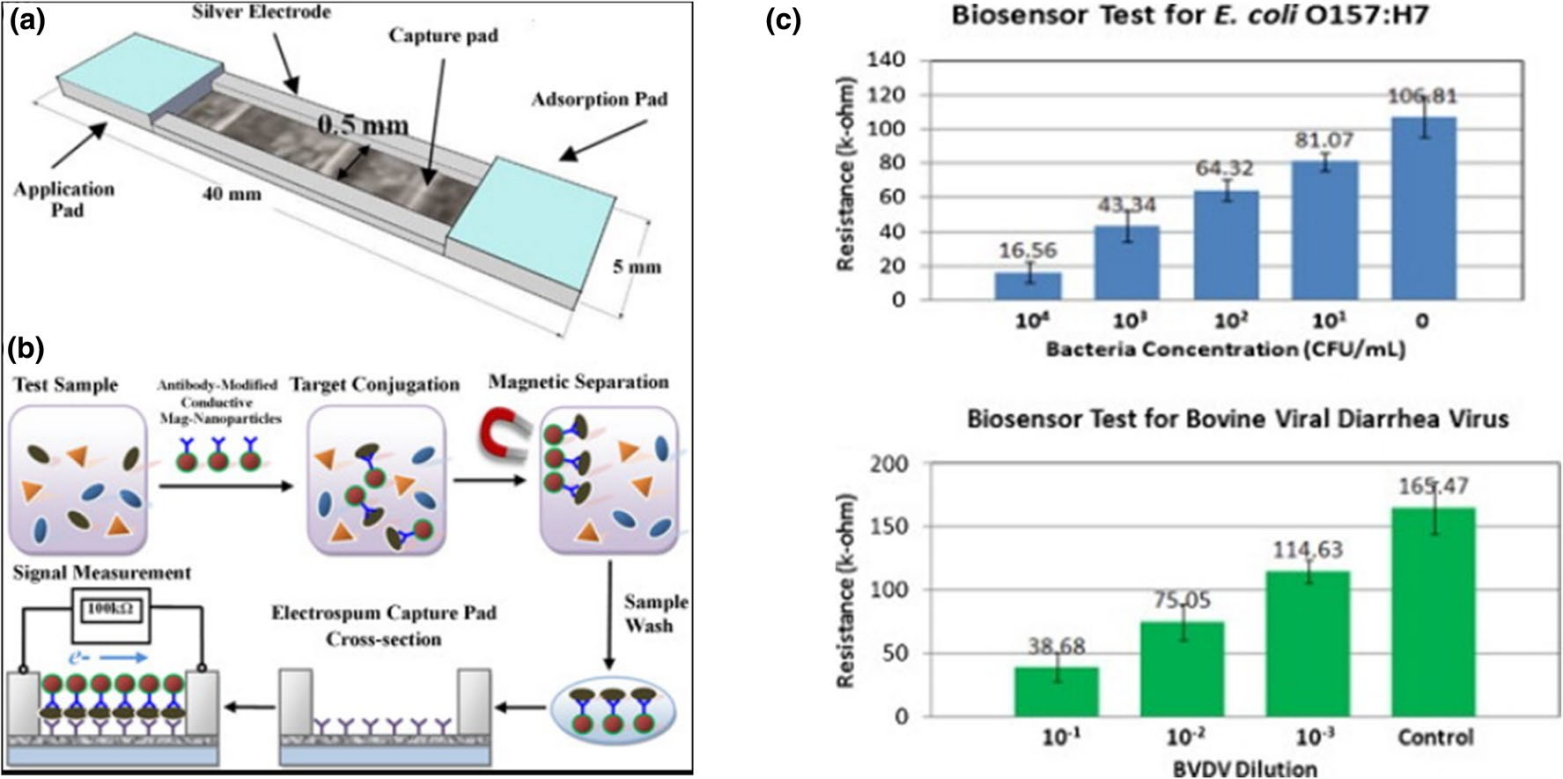
**A** Diagram of the biosensor structure and membrane assembly made up of electrospun cellulose nitrate capture pads and cellulose application and absorption pads. **B** The lateral flow immunosensor's detection strategy based on an electrospun membrane with an antibody functionalization. **C** Biosensor test for *E. coli* and *BVDV* Reprinted from, Ref. [77] with permission from Elsevier Biosensors and Bioelectronics

For the detection of *Escherichia coli (E. coli)* O157:H7 bacteria and *bovine viral diarrhea virus (BVDV),* Luo et al. developed a nanofiber-based biosensing approach [76, 77]. Electrospinning and nitrocellulose porous membrane formation of three different patches, used for application, capture and absorption, provides a lateral-flow immunobiosensor structure (Fig. 5). High surface area and porosity structures played an important role in the capture of and selectivity of microbials. Also, this electrospun nanostructure created a linear response upon exposure to various antigen concentrations. The capture mat was biochemically functionalized by applying *E. coli*. This cutting-edge biosensor required 8 min to detect E. coli O157:H7 bacteria with all the different mats at relatively low concentrations of 67 CFU mL^−1^. The test findings show a linear sensing response range of 10^1^–10^4^ CFU mL^−1^, which is greater than that achieved using a nitrocellulose porous surface under the same conditions. This fast-acting biosensor is made with electrospun technology and is sensitive and affordable.

Paul et al. have demonstrated an ultrasensitive nanobiosensor detection platform with a detection limit of 6.8 ag mL^−1^ for Histidine-rich protein-2 [78]. This nanobiosensor architecture was made up of copper (Cu)-doped ZnO nanofibers that have been electrospun and functionalized with mercaptopropylphosphonic acid (MA). The complementary actions of MA and Cu doping in ZnO are responsible for ultrasensitivity. The functional groups were necessary for immobilizing an antibody are improved by MA. Cu doping in ZnO both increases the conductivity of the nanocomposite and focuses the target component on the MA-treated nanofiber surface because of the intrinsic electrostatic potential formed at the Cu/ZnO hybrid interface. A Cu-doped ZnO nanofiber-based electrode has greater sensitivity (28.5 kΩ (gm^−1^ ml^−1^) cm^−2^) in the detection ranges of 10 ag mL^−1^ to 10 μg ml^−1^. Furthermore, even in the presence of diverse nonspecific molecule interference, the suggested biosensor exhibits strong HRP2 protein selectivity. Additionally, this biosensor exhibits the minimum limit of *malarial* parasite detection that has been documented in literature across a variety of nanomaterials and detection techniques. With a small adjustment, the nanobiosensor platform may be expanded to provide point-of-care diagnostic devices for a number of significant biomarkers because it is based on the immunoassay approach.

To identify the hepatitis B virus (HBV), Niri et al. created a DNA biosensor based on carbon nanofibers (CNFs) [79]. In this work, electrospun CNFs were employed directly as an electrode due to the high electrical conductivity and surface area of CNFs, which make them excellent materials in electrochemical biosensors. The linear range of the DNA measurement was 1 × 10^–12^ to 1 × 10^–6^ M, with a detection limit of 1.58 × 10^–12^ M. The created biosensing platform that worked electrochemically to detect HBV is stable, repeatable, and selective enough to distinguish between complementary and non-complementary DNA sequences.

### Cancer and Cardiac Biomarker

Early detection of cardiological and cancerous conditions allows for quick treatment and prevention of disease progression. With the use of different biomarkers found in the human body, biosensors can rapidly diagnose such serious disorders. Biosensors are utilized to investigate drug interactions with the chosen body regions as well as to identify diseases. For this reason, it is important for the biosensor device used to be reliable, fast-responsive, and cost-effective in terms of reaching patients [80].

Nanofibers produced via electrospinning from PEDOT, poly(4-styrenesulfonate) (PSS) and PVA composite have been investigated to present an electrochemical biosensor instrument. This hybrid nanofiber interface was developed for the recognition of the carcinoembryonic antigen, a cancer biomarker [81]. This structure is both affordable and environmentally friendly. According to the amperometric results, the linear detection range of this nanofiber-based biosensor is 0.2–25 ng mL^−1^ and its sensitivity is 14.2 μA ng^−1^ mL cm^−2^. This functionalized conductive paper electrode offers a viable replacement for creating smart point-of-care devices. And it constitutes a potential example for the recognition of various diseases.

Matta et al. reported that three significant human cardiac biomarkers (Myoglobin, cardiac Troponin I and creatine kinase MB) might be detected using label-free nanofiber-based biosensing that is made up of a single nanofiber that comprised multi-walled carbon nanotubes (MWCNTs) encapsulated in SU-8 photoresist [82]. With the help of electrospinning, these nanocomposites were assembled and following the forming of nanofibers, single nanofibers were positioned between two electrodes. Due to its quick reaction time, high sensitivity, and strong specificity, this MWCNTs embedded SU-8 nanofibrous mat-based biosensor platform offers tremendous potential in the detection of cardiac indicators and other bioanalytes.

### Glucose and Urea

An electrochemical biosensor produced from nanocellulose fibers created through electrospinning was developed to measure the glucose concentration in human blood. It has been stated that this analytical nanostructure, which has high sensitivity and selectivity, also improves analytical performance efficiency using it with rGO. It has been reported that celulose nanofibers (CNs) are also beneficial in separating blood plasma and serum when glucose is loaded. Electrochemical paper-based analytical instruments (ePADs) show high sensitivity and selectivity [83]. The immobilization of glucose oxidase on a layer of freshly prepared CNs was successfully demonstrated. 9.9 × 10^–4^ kΩ^−1^ mM^−1^ was the high sensitivity measured attained by the ePAD glucose test, 0.1 mM to glucose in the range of 3.3–27.7 mM (*R*^2^ = 0.99), very significant repeatability (RSD = 0.57–1.59%), exceptional specificity, and durability.

The completely flexible microfluidics-integrated glucose sensor patch discussed by Bae et al. consists of a stretchable passive microfluidic system and nanoporous gold (NPG) biosensing platform [84]. In order to provide structural elastic properties, high sensitivity, and stability in non-enzymatic glucose biosensing applications, an NPG electrode with a high electrocatalytic activity was constructed on a polydimethylsiloxane (PDMS) membrane. The flexible textile fabric was employed to create a thin and strong microfluidic device that collects and precisely delivers sweat from the skin to the electrode surface. The device was made by inserting the cotton fabric into a thin, PU nanofiber developed PDMS channel that functioned as a capillary. It has been proven that the integrated glucose sensor patch is an excellent instrument for continuously and accurately monitoring sweat glucose levels.

Polyacrylonitrile (PAN) nanofibrous membranes covered with conductive PPy mats were used for forming nanofibrous membranes [85]. On the PAN/PPy/PPy3COOH/ glucose oxide (GOx) impedimetric biosensor response, the impact of various factors was examined. The biosensor response in the optimum test circumstances was linear and shows a wide operating range of glucose concentrations, ∼ 20 nM to 2 μM were measured. The biosensor was also found to have high selectivity features for ascorbic and uric acids.

## Design of Nanofiber-Based Biosensors

### Wearable Biosensors

Wearable biosensors are one of the designs that have recently been of significant importance for the monitoring of human health. These biosensors, which are implanted non-invasively in the human body in various ways, are electronic devices that can simultaneously detect/record data and can be examined by both the patient and the physician [86]. These devices are placed in many different areas of the human body such as tattoos [87], lenses [88], and oral [89], and allow the detection of body fluids/variables such as temperature, saliva, or tears in the human body. Wearable biosensors play a very important role in improving health management and offering the fastest, most reliable, and cost-effective methods of human health with innovations [5, 86].

Electrospinning is majorly utilized in the manufacturing of wearable biosensors due to its well-practiced operation and the numerous benefits of electrospun nanofibers. However, other methods such as pressurized gyration and nozzle pressurized gyration can offer strong alternatives. Wearable biosensors have progressed quickly in recent years, and they have greatly benefited from the development of nanomaterials produced using electrospinning technology. Nanomaterials have also progressed from one-dimensional nanofibers to two-dimensional nanosheets from zero-dimensional nanoparticles. Due to their exceptional qualities such as high surface area to volume ratio, controllable structure and cost-effectivity; nanofibrous mats are often preferred by a variety of industries [5].

**Fig. 6:**
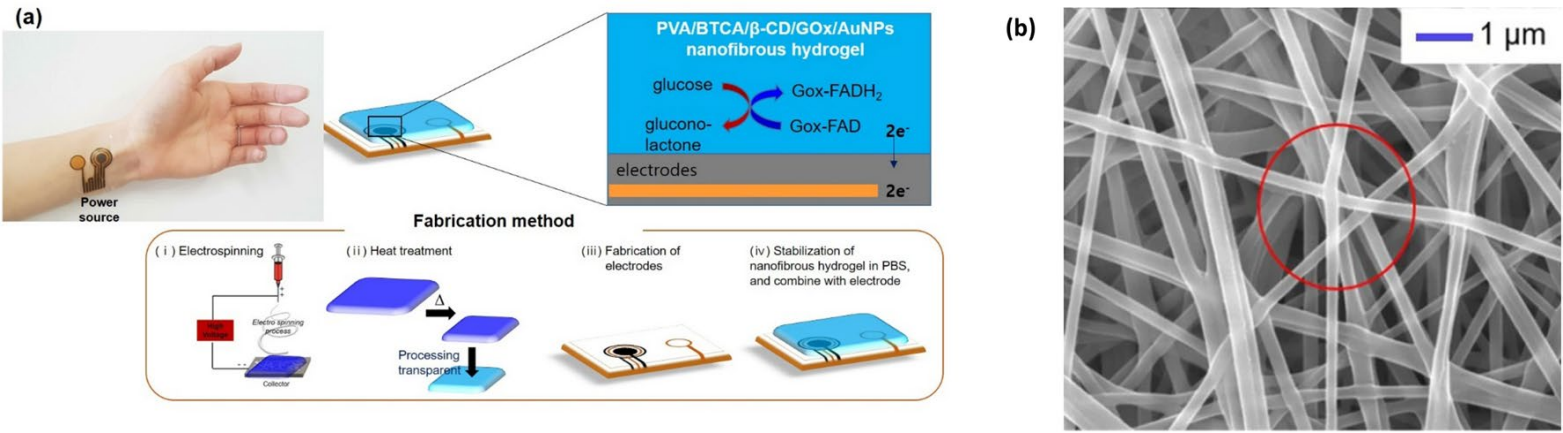
**a** A non-invasive continuous monitoring biosensor fabrication method using electrospinning to applying an electrode for glucose sensing in sweat, **b** SEM characterization of nanocomposite PVA/BTCA/β-CD/GOx/AuNPs hydrogel nanofibers (poly(vinyl alcohol)(PVA), β-cyclodextrin polymer (β-CD), glucose oxidase (GOx), Gold nanoparticles (AuNPs), phosphate-buffered saline (PBS)) Reproduced from Ref. [90] with permission from the Scientific reports Copyright (2020)

Researchers have used micro and nanosized hydrogels produced by the electrospinning technique to produce wearable biosensors. (Fig. 6) The non-invasive biosensing platform contained poly(vinyl alcohol)(PVA) and β-cyclodextrin polymer (β-CD) [90]. AuNPs were added to improve biosensing performance and resulted in high permeability. This biosensor design has shown very encouraging results such as high sensitivity, low sensing limit, and rapid response time. (47.2 μA mM^−1^, 0.01 mM, < 15 s, respectively). According to the findings, the nanofiber-based biosensor has enormous potential for use in clinical settings because it can assess the level of glucose in human serum [90].

For non-invasive, continuous monitoring of an individual's variable health condition, wearable biosensors with high sensitivity to sweat composition analysis are highly desirable. Construction of the mechanically flexible and conductive sensing membrane, which will act as a working electrode for the electrochemical biosensing instruments, is still a challenging task. Wei et al. indicated that a conformal sweat biosensing device based on a fiber-structured sensing surface can detect uric acid. The many active sites of the directed graphitized layer of electrospun carbon nanofibers allow for effective electron transmission while also providing plentiful access to uric acid molecules. The wearable sensing tool created possesses high specificity and selectivity and has the ability to assess the quantity of uric acid in synthetic sweat due to these valuable design features [91].

### Implantable Biosensors

Researchers have made significant strides in recent years toward finding solutions to the issues posed by implantable sensors. There have been significant attempts made to create non-invasive techniques, such as optical and electrochemical biosensing platforms, to quantify the amount of glucose in sweat and skin interstitial fluid. When compared to implanted sensors, non-invasive wearable sensors fall short in terms of dependable and steady long-term functioning performance. For an implantable biosensor to increase its long-term durability and precision, an external membrane must be created [92].

Fang et al. developed a platform with a nanofibrous mat interface that can provide continuous monitoring of glucose levels with a subcutaneously implanted biosensor. The nanofiber composites contained PU and PANi polymers. This structure with high sensitivity (63 nA mM^−1^) and linearity (0–20 mM) was investigated in vitro and in vivo while the change in sensor sensitivity with time was examined. It was observed that it was stable after increasing reactions for the first 2 weeks. The stable observations suggest that this material can be used to monitor glucose in the blood continuously [93].

Researchers presented evidence supporting the use of microporous PVDF membranes sandwiched between several layers of nanoparticles for in vivo continuous glucose monitoring. This was accomplished through layer-by-layer deposited porous layers and covering needle electrodes with PANi nanofiber, platinum nanoparticles, glucose oxidase enzymes and other materials. High surface area and glucose enzyme electrocatalytic activity were produced by nanoparticles integrated into the conductive PANi nanofibers. During the first 7 days of continuous monitoring, the sensitivity was demonstrated to hold within 10% of the original value and stayed at 70% of the initial sensitivity after 21 days [94].

## Current Limitations and Future Applications

Current nanofiber-based biosensors target antibody capture, bioanalyte concentration, analysis signal amplification and have suitable capillary qualities for integration into paper-based devices. Electrospun nanofibers have also shown tremendous potential for the development of improved point-of-care devices. Most of the research show that the electrochemical properties of nanofiber-based biosensors are utilized. These results highlight the fact that nanofiber-based optical and mechanical biosensors also need to be investigated more.

Paper-based biosensors (PPB) have received considerable attention for the development of point-of-care devices because of their simplicity, affordability, and use [95]. In the creation of PPB strips, the use of nanoparticles as labels is essential. The selection of nanoparticles and the accompanying detection technique directly affect the performance of these devices [62, 96]. In addition, viral detection of nanofiber-based biosensors has become a crucial research topic since the COVID-19 pandemic [96]. The cost-effectiveness and easy access of these biosensors help quicker diagnosis.

Very significant of interest has been shown for graphene-based nanomaterials (GNMs), such as graphene, graphene oxide (GO), reduced graphene oxide (rGO), and graphene quantum dots (GQD), in research and industrial applications. GNMs can be used to create a variety of innovative biosensors with enhanced functions and analytical capabilities, providing the possibilities for point-of-care biosensors, lab-on-chip devices, wearable, and flexible electronics [22, 58, 61]. In addition to their large surface area, compact size, physiochemical characteristics, high efficiency of reaction, binding ability, regulated shape and structure, biocompatibility, and electrocatalytic capabilities, GNMs transducers are desirable for a number of reasons [97]. GNMs are ideal electrode materials for creating a variety of sensing platforms due to their structural advantageous and compositional synergy [1]. Particularly, the combination of GNMs and electrochemical biosensors has led to the development of numerous inventive biosensing platforms for use in the field of clinical diagnosis.

However, in practice, commonly used carbon nanomaterials have weaknesses. For example, carbon nanotubes have a propensity to curl and stick together, graphene nanosheets can stack between layers, and carbon quantum dots have a tendency to aggregate and lose their nanostructure properties. Another important restriction on the use of biosensors in the field of electrochemistry is the dispersibility of carbon-based nanomaterials in the solvent. Technical challenges include production costs, purifying issues, and controlled synthesis which currently needs more research. Future studies will also require the application of more advanced scientific techniques to fully comprehend the carbon nanostructures' catalytic mechanism in the redox reaction sensor [98].

Other limitations of electrospun nanofibers are their brittleness and inability to fully adhere to the substrate surface after coating with metal oxide. Nanofibers containing metal oxide can become fragile and break after calcination [99]. This fragility will especially limit the use of a flexible biosensor [100]. In addition, the communication between a nanofiber mat poorly adhered to the electrode surface and the electrode surface decreases and the transmission of the signal becomes weak. This limits the response time and reliability of the biosensor.

## Conclusive Remarks

Biosensing platforms have been developed as a result of the need for precise and quick identification of different substances in a given sample. As research has advanced, the need for such designs has altered as the need for more sensitive and focused determination methodologies has increased. The use of nanomaterials enabled the development of sensing devices with higher loading capacities, quicker response times, better properties and consequently better performance. Nanofibrous membranes have received particular interest in the design of biosensors because of their characteristics including having a high surface area, simplicity in functionalization, and ease of production. Enzymes, microorganisms and cancer biomarkers can all be detected using electrochemical and optical nanofiber-based biosensors. As a result, they have practical uses, such as in point-of-care applications and biomedical devices.

## Data Availability

Data sharing is not applicable to this article as no new data were created or analyzed in this study.
